# Supplementary material for: A deeply supervised adaptable neural network for diagnosis and classification of Alzheimer’s severity using multitask feature extraction
Source: PLoS One. 2024 Mar 26;19(3):e0297996. doi: 10.1371/journal.pone.0297996 (PMC10965088; doi:10.1371/journal.pone.0297996)
Supplement: Attachment — Submitted filename: Review Recommendation PLOS Journals.docx [file pone.0297996.s001.docx]

Review Recommendation:

General identifications: This research explores the application of machine learning (ML) techniques, particularly deep learning, in the early diagnosis and severity classification of Alzheimer's disease using MRI images.

Alzheimer's disease, the most prevalent form of dementia, poses significant challenges due to its gradual progression and varying severity levels. However, recent advancements in neuroimaging techniques have paved the way for large-scale multimodal neuroimaging data, sparking increased interest in leveraging deep learning for automated Alzheimer's disease classification.

The study employs a hybrid approach that combines 12 feature extraction methods to diagnose the severity levels of Alzheimer's disease. In addition, six traditional machine learning algorithms are applied, including decision tree, K-nearest neighbor, linear discrimination analysis, Naïve Bayes, support vector machine, and ensemble learning methods. Each classifier is optimized during training to obtain the best solution.

Notably, the researchers also train a convolutional neural network (CNN) model using a machine learning system algorithm to identify specific patterns in the MRI images. The CNN architecture achieves an impressive accuracy of 95.3%, surpassing the performance of other traditional machine learning methods employed in the study.

Recommendations

1. Dataset Size and Diversity: Use of ADNI and Compare with 4 class of image.
2. This diversity enhances the generalizability of the findings and ensures that the models are not biased towards a specific population.
3. Clinical Validation: While accuracy rates are provided for the different classifiers, there is no discussion on the clinical validation of the proposed approach. It would be valuable to know if the results align with clinical observations and if the proposed method has been tested on real patients to validate its effectiveness in real-world scenarios.
4. Address Interpretability XAI adaptability: Deep learning models, such as CNNs, are known for their black-box nature, which hampers interpretability. The study does not address the interpretability of the CNN model and does not discuss how the model arrived at its predictions. Understanding the underlying decision-making process is crucial, especially in a medical context where interpretability is vital for gaining insights into disease mechanisms.
