# Supplementary material for: A deeply supervised adaptable neural network for diagnosis and classification of Alzheimer’s severity using multitask feature extraction
Source: PLoS One. 2024 Mar 26;19(3):e0297996. doi: 10.1371/journal.pone.0297996 (PMC10965088; doi:10.1371/journal.pone.0297996)
Supplement: Attachment — Submitted filename: Response_to_reviewers_Final.docx [file pone.0297996.s002.docx]

**Response to Respected Reviewers**

We wish to express our sincere appreciation to the esteemed editor and all respected reviewers for providing valuable suggestions and comments. Indeed, these comments led to presenting a better work that has the capability of absorbing more audiences. We revised and improved our paper according to the respected editorials and reviewers’ comments. All the modifications were highlighted in the revised version. Moreover, we have responded to all of the comments in the text below.

**Answer to Reviewer #1:**

*“General identifications: This research explores the application of machine learning (ML) techniques, particularly deep learning, in the early diagnosis and severity classification of Alzheimer's disease using MRI images. Alzheimer's disease, the most prevalent form of dementia, poses significant challenges due to its gradual progression and varying severity levels. However, recent advancements in neuroimaging techniques have paved the way for large-scale multimodal neuroimaging data, sparking increased interest in leveraging deep learning for automated Alzheimer's disease classification.*

*The study employs a hybrid approach that combines 12 feature extraction methods to diagnose the severity levels of Alzheimer's disease. In addition, six traditional machine learning algorithms are applied, including decision tree, K-nearest neighbor, linear discrimination analysis, Naïve Bayes, support vector machine, and ensemble learning methods. Each classifier is optimized during training to obtain the best solution. Notably, the researchers also train a convolutional neural network (CNN) model using a machine learning system algorithm to identify specific patterns in the MRI images. The CNN architecture achieves an impressive accuracy of 95.3%, surpassing the performance of other traditional machine learning methods employed in the study.”*

**Recommendations**

1. Dataset Size and Diversity: Use of ADNI and Compare with 4 class of image.

Thank you for your constructive feedback. We understand the importance and value of integrating widely accepted and recognized datasets such as ADNI for benchmarking. Our chosen dataset presents a diverse range of severity levels for Alzheimer's disease, offering a unique opportunity to test our machine learning models in various scenarios. Such diversity allows us to verify the adaptability and reliability of our methods across different severity stages, which might not be as pronounced in other datasets. The scope of this research, the dataset we used was immediately accessible and suitable for our intended analyses. Incorporating another dataset like ADNI would require adjustments in preprocessing, feature extraction, and potentially model tuning, which could extend the timeline and complexity of our study. While our dataset provides insights into the application of ML methods on MRI images of diverse severity levels, we acknowledge that using ADNI would allow for benchmarking against more established results in the community. Nonetheless, our current experiment holds value as it offers preliminary insights and sets the foundation for further explorations. By validating our methods on this dataset first, it gives us a steppingstone to then branch out and validate against other datasets, including ADNI. In light of the above, we believe our current experiments and findings provide valuable contributions to the field. However, we genuinely acknowledge the potential advantages of integrating the ADNI dataset. In our future endeavors, we plan to not only incorporate ADNI but also discuss in-depth the comparative results, enhancing the robustness and generalizability of our findings.

2. This diversity enhances the generalizability of the findings and ensures that the models are not biased towards a specific population.

Thank you for underscoring the value of dataset diversity. Indeed, the inclusion of diverse data is a cornerstone for building robust models, especially in the realm of medical imaging. By utilizing a varied dataset, we are not only aiming for broader applicability but also safeguarding against inadvertent biases that might arise from narrower data sources. This diversity stands as a testament to our commitment to ensuring our findings are not only accurate but also widely relevant. In future iterations of our work, we will continue to prioritize and expand upon this diversity, further refining our models with even more varied datasets to strengthen the universal applicability of our results.

3. Clinical Validation: While accuracy rates are provided for the different classifiers, there is no discussion on the clinical validation of the proposed approach. It would be valuable to know if the results align with clinical observations and if the proposed method has been tested on real patients to validate its effectiveness in real-world scenarios.

Thank you for emphasizing the importance of clinical validation in real-world scenarios. We recognize its significance in bridging the gap between machine learning research and practical clinical utility. Our methodology, as it stands, was developed and validated against prior literature and datasets. The accuracy rates we've provided offer a comparative measure against previous machine learning endeavors in this domain. Moreover, the MRI images and their corresponding severity levels used in our study are rooted in clinical observations. Therefore, even without direct real-world patient testing, the classifiers' results can be seen as indicative of their potential clinical utility. Also, high accuracy rates, especially across multiple classifiers, hint at a level of diagnostic consistency. This consistency is essential for any tool that could potentially be adopted in clinical settings.

Building upon our findings, it is imperative to subject our approach to rigorous clinical validation. Collaborating with medical professionals will provide invaluable feedback and fine-tuning opportunities. This will ensure that our methods not only retain their accuracy but also resonate with the practical nuances and complexities of real-world clinical scenarios. It remains a top priority for our next phase of research.

4. Address Interpretability XAI adaptability: Deep learning models, such as CNNs, are known for their black-box nature, which hampers interpretability. The study does not address the interpretability of the CNN model and does not discuss how the model arrived at its predictions. Understanding the underlying decision-making process is crucial, especially in a medical context where interpretability is vital for gaining insights into disease mechanisms.

Thank you for highlighting the vital aspect of model interpretability, especially in the sensitive domain of medical diagnosis. We completely understand that the powerful performance of CNNs often comes with the drawback of reduced transparency. In clinical applications, the trust clinicians place in a diagnostic tool is fundamentally based on understanding the rationale behind its predictions. To offer some insight into our model's operations, we've visualized the intermediate layers of the CNN to gain insights into feature activations. These visualizations can help elucidate what the network emphasizes or prioritizes during the decision-making process. Additionally, we've employed saliency maps to highlight crucial regions in the MRI images that the model deems significant for classifying different severity levels, providing a visual representation of brain regions influencing the model's decisions.

However, we recognize the depth of the interpretability challenge and the necessity of addressing it more holistically. We're actively looking into integrating advanced eXplainable AI (XAI) techniques, to offer granular, instance-specific explanations for our model's predictions. By comparing these explanations with expert clinical feedback, we aim to ensure that the decision-making process of our model is not only transparent but also medically sound. We believe that the integration of XAI methods will enhance the trustworthiness of our approach, potentially offering fresh insights into disease mechanisms and guiding subsequent research. Rest assured, enhancing the interpretability of our CNN model is a priority in our ongoing and future endeavors.

5. English proofread is needed over again.

Thank you for pointing this out. We apologize for any oversights in the initial submission and have ensured a thorough proofreading and language review to enhance the clarity and coherence of the manuscript. Your feedback is invaluable in improving the overall quality of our research paper.

**Reviewer #2:** To be honest, the author did a comprehensively good job in this regard. However, I have very little concern:

1. For the title to have "deeply and deep" together for me I believe it can be coined in a different way.

We appreciate your feedback on the title's structure. We strive for clarity and coherence, and understand that the repeated usage of terms like "deeply" and "deep" can lead to redundancy. We revisited the title and aimed for a more succinct representation that captures the essence of the study.

2. The introduction and literature review were quite ok but, I suggest for both sections to be seen broader and add real value, covering some useful studies which will interest reader would advisable. For instance.

- A Machine Learning Method with Filter-Based Feature Selection for Improved Detection of Chronic Kidney Disease”
- Integrating Enhanced Sparse Autoencoder Based Artificial Neural Network Technique and SoftMax Regression for Medical Diagnosis MDPI Electronics Journal,
- An Interpretable Machine Learning Approach for Hepatitis B Diagnosis.

Thank you for finding the introduction and literature review satisfactory. We recognize the value of broadening our literature review to provide a more comprehensive understanding of the domain. We have certainly incorporated the studies you mentioned. Including these will undoubtedly make our paper richer and cater to a broader audience.

As follows: “By combining information-gain-based feature selection with an AdaBoost classifier, Ebiaredoh-Mienye et al. (2022) highlighted the effectiveness of ML in early Chronic Kidney Disease (CKD) identification. Earlier, Ebiaredoh-Mienye et al. (2020) used an improved sparse autoencoder with Softmax regression to address unbalanced medical datasets, resulting in superior disease prediction outcomes for CKD, cervical cancer, and heart disease. While employing ML models in conjunction with SHapley Additive exPlanations (SHAP) for greater interpretability, Obaido et al. (2022) improved hepatitis B diagnosis, underlining the value of certain characteristics like bilirubin in predicting outcomes.”

3. The Methods and Materials section was very understandable based on the algorithms proposed and adopted at the end. In figure 1,2,3, I believe the feature extraction, the author should also look at other novel methods which will go along way to assist in terms of insight (“A Neural Network Ensemble with Feature Engineering for Improved Credit Card Fraud Detection” IEEE Access, Vol 10, pp.16400- 6407, 2022) and (“Sparse Noise Minimization in Medical Image Classification Using Genetic Algorithm and DenseNet” IEEE International Conference on Information Communication Technology Society, pp.10-11, March 2021, Durban, South Africa. DOI: 10.1109/ICTAS50802.2021.9395014).

Thank you for your comments. We added your valuable paper to the method section as follows: Advanced machine learning and optimization approaches have demonstrated intriguing uses in recent medical research. As an illustration, consider an ensemble strategy that uses LSTM neural networks and hybrid data resampling to optimize fraud detection or medical imaging that uses genetic algorithms to improve picture accuracy before utilizing DenseNet for classifications.

4. If the authors can they should look at sensitivity, and precision and possibly and F1 score as some indices/metrices in there work. I see they touched on accuracy which is very ok.

Thank you for your comments.
