# Supplementary material for: A deeply supervised adaptable neural network for diagnosis and classification of Alzheimer’s severity using multitask feature extraction
Source: PLoS One. 2024 Mar 26;19(3):e0297996. doi: 10.1371/journal.pone.0297996 (PMC10965088; doi:10.1371/journal.pone.0297996)
Supplement: Attachment — Submitted filename: Response to Reviewers.docx [file pone.0297996.s003.docx]

**-Response to Editor and Reviewers-**

We wish to express our sincere appreciation to the esteemed editor and all respected reviewers for providing highly valuable suggestions and comments. Indeed, these comments led to presenting a better work that has the capability of absorbing more audiences. We have carefully revised and improved our manuscript according to the respected reviewers' comments. All the new modifications have now been highlighted in the revised manuscript. Besides, we have responded to all the comments in this response letter.

**Reviewer #1: All comments have been addressed.**

Response: We truly appreciate your time and efforts.

**Reviewer #3:**

**Comment 1.** The title is not attractive and should be re considered.

**Response:** Thank you so much for your comment. The title has now been changed and summarized to "A Deeply Supervised Adaptable Neural Network for Diagnosis and Classification of Alzheimer’s Severity Using Multitask Feature Extraction"

**Comment 2.** It does not clearly show your innovations and contributions. Please highlight your innovations.

**Response:** The novelty and the contribution of the paper have been revised and changed at the end of the introduction section as follows,

“This study utilizes machine learning (ML) techniques to determine the severity of Alzheimer's disease. The dataset used in the study comprises MRI images with four different levels of severity. We used a hybrid of 12 feature extraction methods to diagnose Alzheimer's disease severity using MRI images. Six traditional machine learning methods are used to diagnose Alzheimer's disease. The techniques include decision tree (DT), K-nearest neighbor (KNN), Linear Discrimination Analysis (LDA), Naive Bayes (NB), Support Vector Machine (SVM), and ensembled learning, including Bag, Adaboost, and RUSBoost methods. In the training process, optimization is done to find the best solution for each classifier. Moreover, a CNN model is taught to recognize patterns using a machine learning technique.”

**Comment 3.** I suggest to organize the text in the standard way to include introduction, methodology, results, discussion, and conclusion. The goal of the paper should be better specified.

**Response:** Following the respected reviewer's comment, we updated the paper organization and reflected it in the revised manuscript as follows.

“The remainder of the paper is organized as follows: Section 1 is introduction, Section 2 presents the related work, while Section 3 describes the methods and materials used in the study. Section 4 presents the results of the experiments and a discussion of the findings. Finally, Section 5 provides the conclusion and future work.”

**Comment 4.** The motivation is not clear. Please specify the importance of the proposed solution.

**Response:** Thanks for your comment. We have made modifications to indicate our paper's importance in a clearer manner.

The paper's motivation are highlighted at the conclusion of the introduction section, emphasizing the use of machine learning (ML) techniques to assess Alzheimer's disease severity. The research employs a dataset of MRI images categorized into four distinct severity levels. A combination of 12 feature extraction methods is applied to evaluate Alzheimer's disease severity based on these MRI images. The study utilizes six conventional machine learning methods for diagnosis, including decision tree (DT), K-nearest neighbor (KNN), Linear Discrimination Analysis (LDA), Naive Bayes (NB), Support Vector Machine (SVM), and a combination of ensemble learning techniques such as Bagging, AdaBoost, and RUSBoost. Optimization is a key part of the training process, aiming to discover the most effective solution for each classifier. Additionally, the study involves training a Convolutional Neural Network (CNN) model to detect patterns using machine learning techniques.

**Comment 5.** You should explain this model by using steps.

**Response:** Thank you so much for this accurate comment. We addressed this important comment in the revised manuscript. Indeed, the steps are as follows:

**Step 1- Hybrid Feature Extraction:**

Utilization of a hybrid of 12 feature extraction methods for diagnosing Alzheimer's disease (AD) severity using MRI images.

- Feature Extraction Methods: These include GLCM, LBP, RLBP, LTP, SGM, BIBIS, PCA Filter, ICA Filter, Gabor Filter, Log-Energy, Model-based Feature, and conventional shape signature, as illustrated in Figure 2 and based on Figure 1.
- Total Features: Extraction of a total of 90 image features.

**Step 2- Image Preprocessing:**

Rescaling: Transforming images to double. Grayscale Conversion: Transforming images to grayscale. Normalization: Normalizing the images for consistent processing.

**Step 3- Feature Reduction:**

PCA Method: Implementing PCA feature reduction to decrease computation time and optimize the training process.

**Step 4- Classification:**

Categorizing the output labels into four distinct groups

**Comment 6.** Some papers can be discussed. For example:
- https://doi.org/10.1007/s11571-022-09859-2
- https://doi.org/10.1016/j.compbiomed.2021.104828
- <https://doi.org/10.1016/j.medengphy.2023.103971>

**Response:** Thank you for your suggestions. We have added your reference to the paper as follows, Dogan et al. (2023) developed a model of primate brain patterns based on EEG signals for the detection of AD. Using a directed graph to extract features from the primate brain's connectome, this method demonstrated high accuracy in identifying AD patients from healthy controls. In order to enhance the accuracy of AD detection, the model is able to generate a set of features from EEG signals. Using brain images, Kaplan et al. (2021) developed a feed-forward Local Phase Quantization Network (LPQNet) for AD detection. Based on feature generation and selection through multilevel processing, their model demonstrated remarkable classification accuracy across several datasets. LPQNet stands out for its combination of high accuracy and low computational complexity, which makes it a valuable tool for diagnosing Alzheimer's disease. Kaplan et al. (2023) developed the ExHiF model for the detection of AD using CT and MR images. They combined exemplary histogram-based features with neighborhood component analysis to achieve 100% classification accuracy. In terms of medical image classification for AD, this model is innovative in its feature extraction process inspired by vision transformers. Newly added references have been highlighted in the revised paper, Ref. [23], [24], [25].

**Comment 7.** What is the research gap? How was this research gap filled?

**Response:** The research gap in this study pertains to the inadequacy of existing methods for diagnosing Alzheimer's disease severity using MRI images. Prior approaches may have lacked comprehensive feature extraction, utilization of advanced machine learning techniques, or effective optimization strategies to accurately assess the disease's severity. This study fills this gap by introducing a hybrid approach that combines 12 feature extraction methods and six traditional machine learning techniques, including ensemble learning methods. Moreover, the study enhances the diagnostic process by implementing a Convolutional Neural Network (CNN) trained to recognize complex patterns in MRI images and by optimizing the classifiers to ensure a more accurate and efficient diagnosis of the severity of Alzheimer's disease.

**Comment 8.** Figures and tables should be discussed comprehensively.

**Response:** Thank you for your comment. We have double-checked and revised the descriptions of the figures and tables and provided further explanations.

**Comment 9.** More details about network architecture and complexity of the model should be provided

**Response:** Thank you for your comment. We have added the network architecture and complexity of the model in Figure 2. In this work, six ML methods are employed to diagnose AD. The techniques include DT, KNN, LDA, NB, and SVM, and ensemble learning includes Bag, Adaboost, and RUSBoost methods. In the training process, optimization is carried out to find the best solution for each classifier. DT is the first classifier used in the training process.
